# Low Nephron Number and Kidney Disease

**DOI:** 10.3390/ijms27198888

**Published:** 2026-10-07

**Authors:** Samaneh Goorani, John D. Imig

**Affiliations:** Department of Pharmaceutical Sciences, University of Arkansas for Medical Sciences, Little Rock, AR 72205, USA; sgoorani@uams.edu

**Keywords:** glomerulus, nephron, chronic kidney disease, hypertension, hypoplastic kidney

## Abstract

Reduced nephron endowment and acquired nephron loss increase susceptibility to hypertension and chronic kidney disease (CKD). Low birth weight, prematurity, genetic factors, and adverse prenatal exposures can limit nephron development, whereas aging and kidney injury reduce the number of functioning nephrons after birth. Compensatory glomerular hypertrophy and increased single-nephron filtration can initially preserve overall glomerular filtration rate, but sustained glomerular stress may promote podocyte injury, proteinuria, and glomerulosclerosis. These changes can accelerate nephron loss and create a cycle of progressive kidney damage. Disease susceptibility depends on genetic background and additional metabolic, environmental, and hemodynamic stresses. This review examines the determinants of nephron number, the adaptive and potentially harmful consequences of nephron reduction, and approaches to preserving kidney function. Improving maternal health, reducing harmful exposures, and controlling hypertension and metabolic disease may reduce the long-term consequences of low nephron number.

## 1. Introduction

Nephrons are the functional units of the kidney responsible for filtering blood, producing urine, and maintaining fluid and electrolyte balance [1]. Nephron number contributes to kidney functional capacity, but varies considerably among individuals [2,3]. Low nephron endowment at birth and subsequent nephron loss increase susceptibility to hypertension and chronic kidney disease (CKD). Nephron loss also accompanies normal aging and contributes to the age-related decline in glomerular filtration rate (GFR) [2,3].

In animal models of kidney disease, nephron loss begins due to damage that occurs outside the glomerular capsule [4]. In this process, podocyte cells play an important role. If injuries are confined within the endocapillary compartment, they will either be repaired by scarring or undergo recovery. Dysregulation, inflammatory, and degenerative mechanisms that result in nephron loss are differentiated [5,6]. The dysregulated mechanisms that result in collapsing glomerulopathy are distinctive in that they affect glomeruli and tubules in parallel, in addition to several other unique features [7]. Conversely, tubular injury is a consequence of glomerular lesions in degenerative and inflammatory diseases. In both latter categories of diseases, the progression commences in the glomerulus with the loss of the separation between the tuft and Bowman’s capsule [8]. This is achieved by the formation of cell bridges (parietal cells and/or podocytes) between the glomerular and parietal basement membranes. Cell bridges transform into tuft adhesions to Bowman’s capsule, which trigger the formation of crescents. This process is initiated by either misdirected filtration (proteinaceous crescents) or epithelial cell proliferation (cellular crescents). Crescents have the potential to extend onto the tubule through the glomerulotubular junction and cover the entire circumference of the glomerulus [9].

Progression can involve increased workload in surviving nephrons and inflammatory or fibrotic responses to injury [10,11]. Compensatory hyperfiltration may initially preserve kidney function, but sustained mechanical stress can promote further glomerular damage. Repair is limited, and segmental glomerulosclerosis can develop at sites of irreversible injury [6,12].

Glomerular and tubulointerstitial matrix accumulation are common features of progressive kidney disease. However, the contribution of fibrosis to injury in otherwise intact nephrons remains incompletely defined [4,11]. Many progressive kidney diseases begin with glomerular lesions, emphasizing the importance of understanding how glomerular injury leads to nephron loss [13,14].

Low nephron number increases single-nephron glomerular filtration, leading to glomerular enlargement, hyperfiltration, and hypertension. Persistent glomerular stress promotes glomerulosclerosis, nephron loss, progressive kidney injury, and microalbuminuria.

## 2. Overview of Nephron Number and Kidney Health

**Figure 1:**
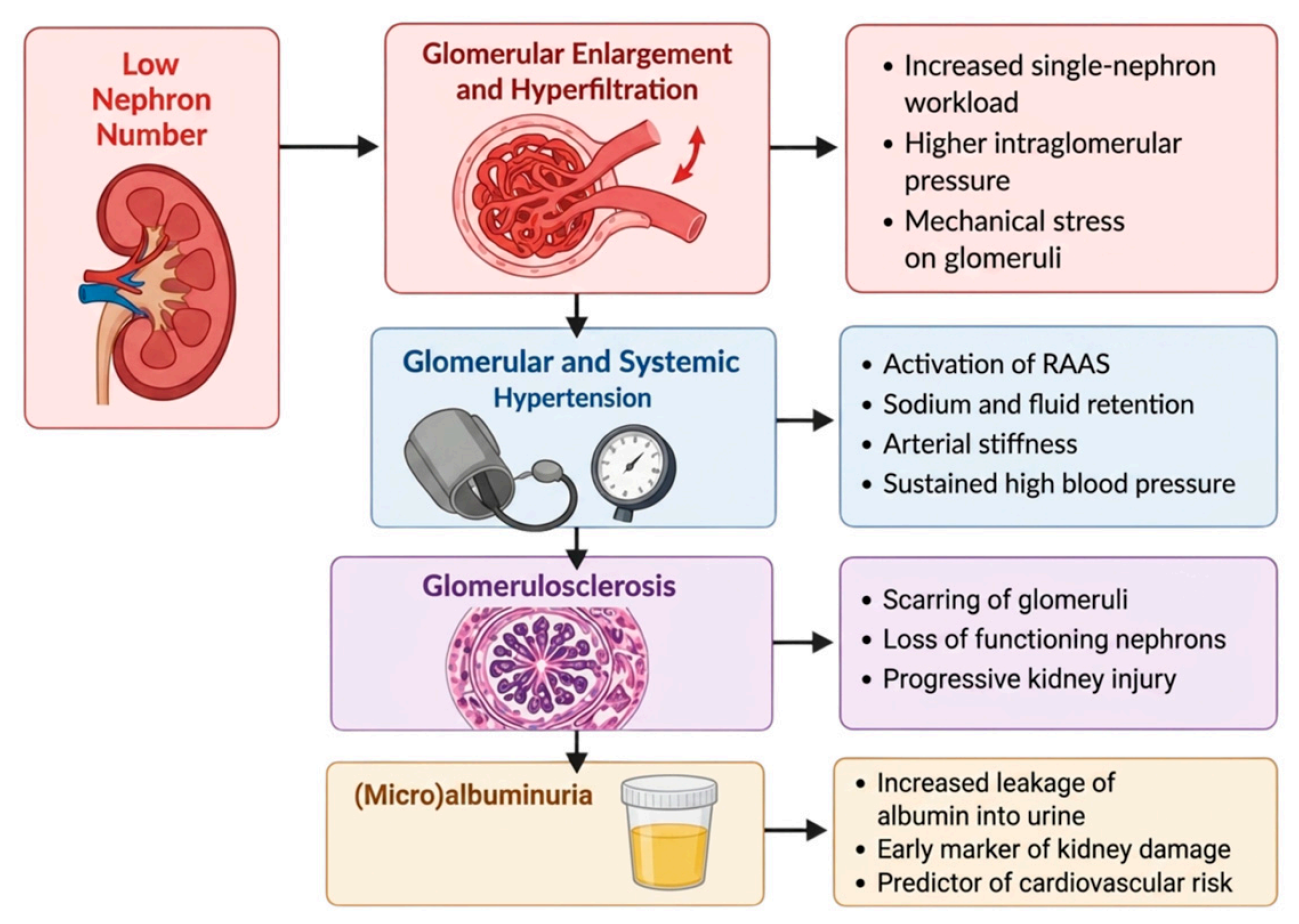
Pathophysiological Consequences of Low Nephron Number.

Nephrons regulate fluid volume, electrolytes, acid–base balance, and waste excretion; these functions depend on both nephron number and structural integrity [15,16]. When nephron number is reduced, the remaining nephrons can enlarge and increase single-nephron glomerular filtration rate (SNGFR), partially preserving overall GFR [17,18,19]. Sustained hyperfiltration and elevated glomerular capillary pressure may subsequently promote glomerulosclerosis and further nephron loss (Figure 1). Thus, a preserved overall GFR does not necessarily indicate a normal nephron complement.

Low nephron number is associated with hypertension and kidney disease, although the relationship varies among individuals [18,20,21]. Hypertension can both accompany nephron deficiency and accelerate additional kidney injury. Monitoring kidney function, albuminuria, and blood pressure in at-risk populations can help identify progressive injury and guide early management of modifiable risk factors [22,23,24].

Genetic, prenatal, and environmental influences produce substantial variation in nephron number, making a universal threshold for kidney dysfunction difficult to define [17,21]. Emerging imaging approaches may improve assessment of this variation. Cationic ferritin-enhanced magnetic resonance imaging has enabled whole-kidney nephron assessment and longitudinal tracking in vivo in rodents, offering a research tool for relating nephron number to kidney function and disease progression [22,25,26].

## 3. Impact of Nephron Number on Kidney Function

Nephron endowment influences lifetime susceptibility to kidney disease. Human autopsy studies report approximately 210,000 to 2,700,000 nephrons per kidney [22,27]. Nephrogenesis is normally completed before full-term birth, and endowment reflects perinatal, genetic, and environmental influences [28]. Prematurity can interrupt this process and is associated with increased risks of acute kidney injury and CKD [29]. The causes of the wide variation in human nephron number remain incompletely understood.

Developmental adaptation to intrauterine stress may favor early survival at the expense of somatic growth and nephron endowment, increasing later susceptibility to CKD [22,30]. Advances in neonatal care allow very premature infants to survive while kidney development continues outside the uterus [31]. Although postnatal glomerular formation has been observed, subsequent kidney health also depends on neonatal injury and later comorbidities, including obesity, diabetes, and hypertension [32,33]. Epidemiologic studies associate preterm birth with CKD, but direct longitudinal measurements of nephron number remain limited [22,33].

## 4. Relationship Between Birth Weight and Nephron Number

**Figure 2:**
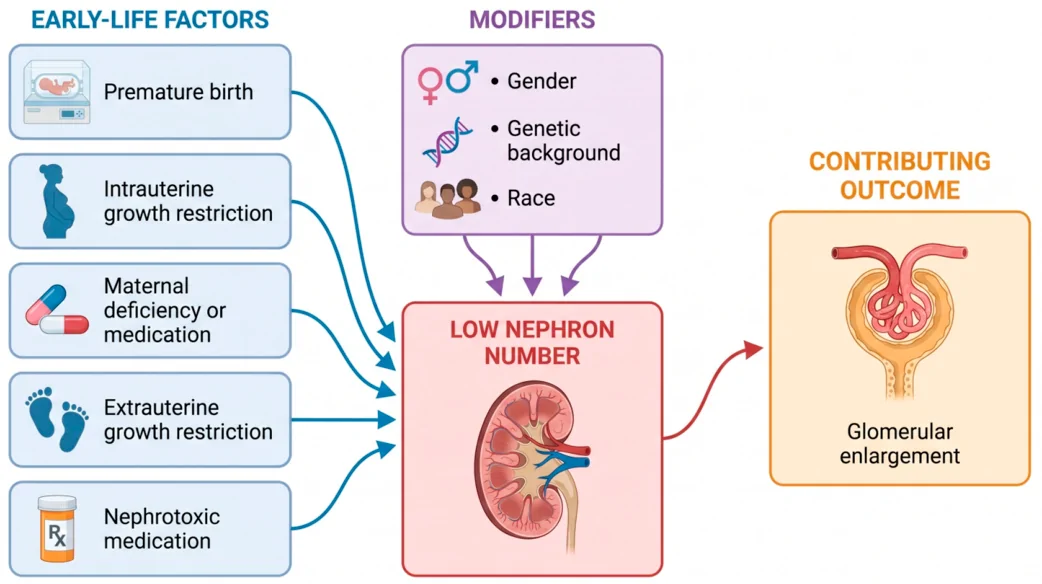
Determinants and Contributors to Low Nephron Number.

Fetal programming is a critical concept that refers to the direct influence of prenatal environmental factors, particularly low birth weight, on kidney development and the risk of nephron-related defects in adulthood [34] (Figure 2). Research shows that a lower number of nephrons, usually caused by adverse prenatal conditions, can lead to long-term health problems, including CKD and heart disease [35]. Environmental conditions during pregnancy can affect the development of the fetus, and these effects can have long-term health consequences [36]. The fetal programming phenomenon is strongly related to the “developmental origins of health and disease” hypothesis, which states that early life exposures can predispose a person to a variety of chronic diseases in adulthood, including kidney disorders [37]. Low birth weight is recognized as one of the significant consequences of intrauterine planning and usually refers to a birth weight of less than 2500 g. Research shows that babies classified as low birth weight are at higher risk of long-term health complications, such as kidney problems [38]. When other factors such as maternal health, nutrition, and gestational age are considered, the association between low birth weight and kidney disorders becomes particularly strong [39].

Nephron development, nephrogenesis, primarily occurs during the prenatal period. Research suggests that the development of nephrons is highly susceptible to environmental factors, and any insults inflicted during pregnancy can have a detrimental impact on nephron numbers [40]. The kidneys depend on an adequate nephron count to regulate fluid and electrolytes efficiently, which is why a reduced nephron count is a risk factor for future hypertension and renal impairment [41]. Reducing the quantity of nephrons can have significant consequences. Hypertension and a higher risk of developing chronic kidney disease and other related complications in maturity are associated with lower nephron counts [42]. The critical need for understanding neonatal programming as it relates to kidney health was underscored by an estimated meta-analysis, which indicated that individuals with low birth weight face an approximately 83% higher risk of CKD in later life [43]. The objective of emerging research endeavors is to comprehend the precise biological mechanisms through which fetal programming influences kidney development, as well as potential interventions that could alleviate the adverse impacts of low birth weight [34]. The increasing recognition that addressing nutritional and health disparities during pregnancy could improve outcomes for future generations and reduce the incidence of adult-onset kidney diseases linked to nephron number deficiencies is gaining momentum [44].

Premature birth, growth restriction, maternal factors, and nephrotoxic medications can reduce nephron endowment, with effects influenced by sex, genetic background, race, and environmental factors. Low nephron number subsequently contributes to compensatory glomerular enlargement.

## 5. Compensatory Mechanisms of Low Nephron Number

Congenital nephron deficiency, nephrectomy, and disease-induced nephron loss trigger compensatory adaptations in surviving nephrons [19,45,46]. Glomerular and tubular hypertrophy increases renal plasma flow, and higher SNGFR can initially preserve overall GFR [2,47,48,49,50]. Children with a congenital solitary functioning kidney may therefore maintain GFR despite reduced renal mass [51]. These adaptations support short-term function, but their long-term consequences depend on the degree of nephron reduction and additional stresses (Figure 1) [18,19].

Persistent hyperfiltration and elevated glomerular capillary pressure can increase mechanical stress on podocytes, promoting injury and detachment [10,52,53]. Loss of podocyte coverage compromises the filtration barrier and can contribute to proteinuria and glomerulosclerosis [54]. Progressive scarring reduces the number of functioning nephrons, increasing the workload of those that remain and reinforcing the cycle of kidney injury. Hypertension and metabolic stress may amplify this process. Monitoring blood pressure, albuminuria, and kidney function is therefore important in people with congenital nephron deficiency or acquired nephron loss [3,18,23,55].

## 6. Environmental Influences on Nephron Number

The development of nephrons during pregnancy is significantly influenced by environmental factors, such as maternal health, nutrition, and exposure to pollutants, which may have long-term health implications (Figure 2). Human research indicates that reduced nephron numbers can be the result of both maternal and lifestyle influences, which are linked to elevated risks of CKD and hypertension in later life [56]. Maternal factors, such as smoking, hypertension, and other stressors, can have a detrimental impact on renal development, which in turn contributes to variations in the number of nephrons across populations, ultimately resulting in substantial health implications. Reduced nephron numbers in offspring are notably associated with maternal diabetes, which can result in complications such as elevated blood pressure and CKD [21]. The nephron count is reduced because of in utero exposure to maternal hypertension, which impairs nephron progenitor differentiation. The significance of managing maternal health during pregnancy to protect renal outcomes in children is underscored by these complications [57].

The development of the nephron is significantly influenced by maternal nutrition. Nephrogenesis can be influenced by both over- and under-nutrition, which can lead to a persistent decrease in the number of nephrons. For example, maternal dietary restrictions during critical periods, such as the second half of gestation, can substantially disrupt the formation of nephrons [58]. The risk of diminished nephron counts is further exacerbated by conditions such as intrauterine growth restriction (IUGR), which is associated with poor nutrition. To achieve optimal fetal kidney development, these results emphasize the importance of appropriate maternal nutrition [59].

It has been demonstrated that exposure to environmental pollutants during pregnancy can have a detrimental impact on nephron development [60]. The formation of the kidney can be adversely affected by prenatal exposure to toxic metals, such as arsenic, cadmium, lead, and mercury, which may result in CKD later in life [61]. Research indicates that these toxic substances have their most detrimental effects during critical stages of neonatal development, thereby diminishing the quantity of functional filtering units in the kidney [62]. Enhanced development outcomes in infants could be achieved by comprehending and mitigating such exposures. Reduced nephron numbers in progeny may result from maternal stress during pregnancy, which induces elevated levels of oxidative stress [63]. Various adverse neonatal outcomes, such as impaired nephron development, have been associated with increased oxidative stress. The argument for a supportive and healthy prenatal environment as essential for optimal kidney health in the subsequent generation is further substantiated by the influence of stress on nephron reduction [64].

Lifestyle factors, including diet and physical activity, can have a substantial impact on the development of nephrons, in addition to maternal health and environmental pollutants [64]. The quantity of nutritional support that reaches the fetus can be influenced by maternal lifestyle choices, such as physical activity levels and dietary habits. For instance, a diet that is deficient in essential nutrients reduces nephron endowment in offspring and increases the risk of low birth weight [65]. Furthermore, the reduction in nephron endowment is further exacerbated by inadequate maternal weight gain and associated lifestyle factors [66]. These discoveries have significant implications for public health, particularly in the context of interventions that foster maternal and reproductive health. Enhancing nephron development and reducing the risk of kidney disease in future generations can be achieved by assuring nutritional adequacy during pregnancy, minimizing exposure to environmental toxins, and improving maternal health outcomes [33,65].

Environmental factors also influence kidney disease progression after birth in individuals with reduced nephron endowment. There have been reports that up to 30% of patients with at least a 50% reduction in renal mass develop hypertension and 70% develop proteinuria, leading to CKD [67]. Experimental studies in mice born with reduced nephron endowment demonstrated an influence of environmental and nutritional factors on kidney health. Hyperlipidemia in ROP *Os*/+ mice born with reduced nephron endowment resulted in a progressive decline in renal function mediated at least in part through increased inflammatory cytokines [68]. Likewise, mice with low nephron number are more susceptible to gentamicin-induced acute kidney injury and at greater risk to develop CKD [29]. This demonstrates that reduced nephron endowment results in greater susceptibility to lifestyle and environmental factors and increases the risk of CKD in this vulnerable population.

## 7. Sex Differences in Nephron Number

Sex differences in nephron number have been reported in humans, but the findings are not consistent across studies. Some investigations indicate that females have fewer nephrons per kidney than males. For example, a 2023 study using kidney imaging and biopsy measurements found that female sex was associated with lower estimated nephron number [69]. However, an earlier stereological study found no significant difference in glomerular number between men and women [70]. Thus, the available evidence supports a possible average difference in some populations, rather than a universal distinction between the sexes.

Differences in kidney size should also be distinguished from differences in nephron number. Larger kidneys do not necessarily contain more nephrons. Neugarten and colleagues found that the larger kidneys and glomeruli observed in men were explained by their greater body surface area, while glomerular number was similar between sexes [70]. Another autopsy study reported approximately 15% fewer glomeruli in females, but this difference was not statistically significant [71]. These findings emphasize the need to account for body size, age, and substantial individual variability when comparing nephron numbers between males and females.

Experimental studies likewise show that larger male kidneys do not necessarily contain more nephrons. In one mouse study, animals receiving a normal-protein diet had nearly identical nephron numbers in males and females, averaging approximately 11,000 per kidney [72]. This finding demonstrates that sex differences in renal growth can occur without corresponding differences in nephron number. These results support measuring nephron number directly and evaluating males and females within the specific experimental model in studies of renal hypoplasia or kidney injury, rather than if females have fewer nephrons because their kidneys are smaller.

## 8. Genetic Factors Determining Nephron Endowment

The nephron number is significantly influenced by genetic determinants, resulting in variability across various populations [22] (Figure 2). Numerous genetic factors influence embryonic development and nephron endowment, which is fixed at full-term birth. Over 400 genes have been identified as influencing kidney development, and nephrogenesis is influenced by a variety of genes [73]. Genetic studies in humans and mice have provided valuable insights into the genetic contribution and molecular mechanisms resulting in reduced nephron endowment [74]. Several interacting signaling pathways, including glial cell line-derived neurotrophic factor to the RET receptor tyrosine kinase via a GFRα co-receptor (GDNF/RET), fibroblast growth factors (FGF), paired box gene 2 (PAX2), and hereditary hemochromatosis (HH), have been strongly implicated in nephrogenesis and low nephron number [75,76]. Continued advances in human and mouse genetic analyses are expected to facilitate the identification of additional known and novel genes involved in determining nephron endowment.

Nephron endowment reflects interactions among genetic, epigenetic, and environmental influences [76,77]. Genetic background also modifies the consequences of nephron deficiency: mouse strains carrying the *Os* mutation can develop different degrees of glomerulosclerosis despite comparable reductions in nephron number [78]. ROP *Os*/+ mice develop glomerular enlargement, increased cellularity, tubular hypertrophy, and progressive glomerulosclerosis, with increased expression of extracellular matrix proteins including collagen type IV and tenascin [78,79]. Nephron-deficient Fvb mice develop rapidly progressive renal failure and marked albuminuria [80]. These models demonstrate that reduced nephron number alone does not fully determine disease severity; genetic susceptibility and compensatory responses also influence progression [78,81].

Defining the genetic determinants of nephron endowment and susceptibility to injury may improve risk assessment. Noninvasive approaches to estimating nephron number and biomarkers of early damage could help relate these determinants to clinical outcomes and support individualized preventive strategies [81,82].

## 9. Effect of Aging on Nephron Number

**Figure 3:**
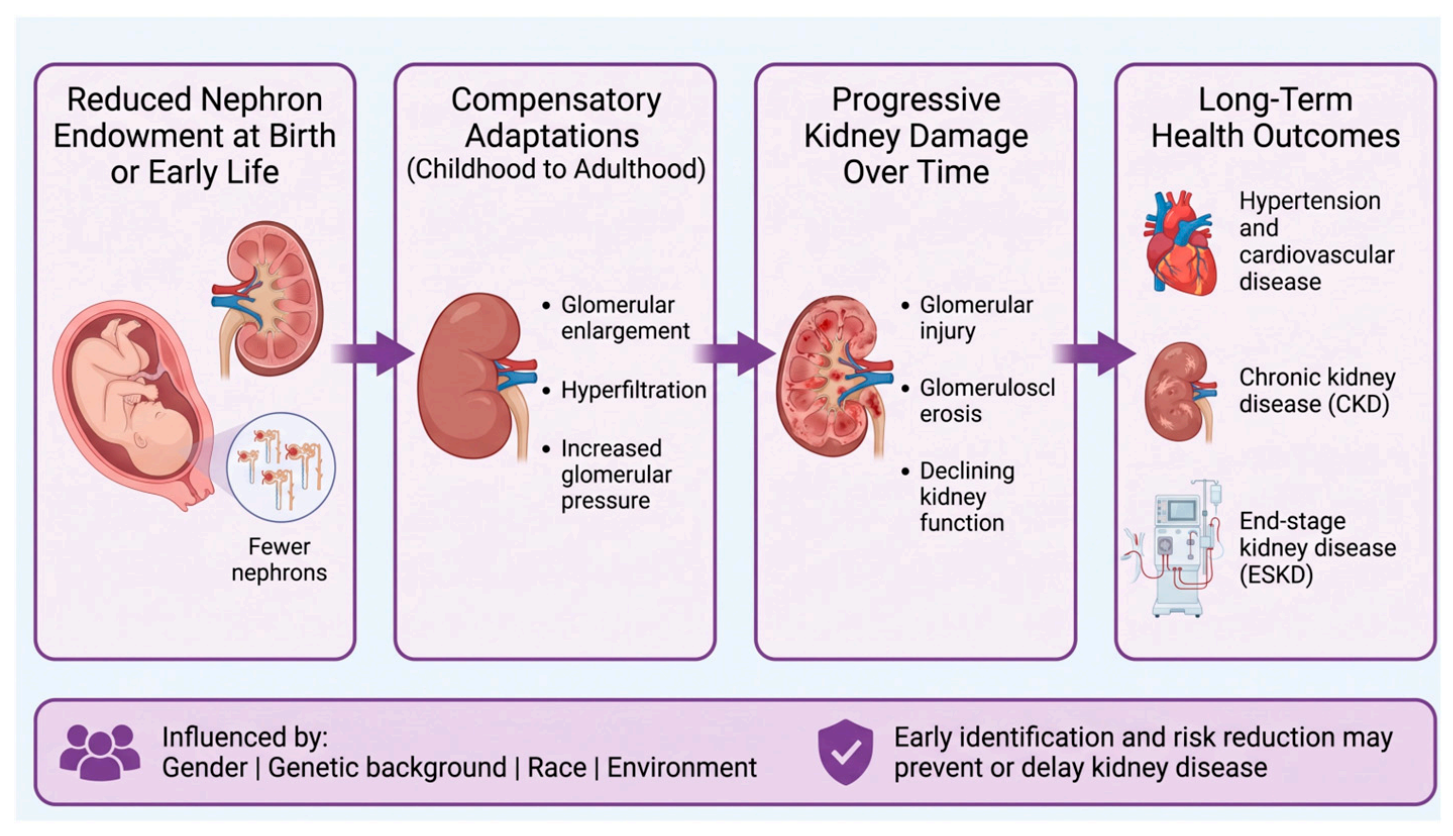
Long-term Impact of Reduced Nephron Endowment.

Significant nephron loss and pathophysiological alterations in the kidneys are associated with aging, which directly impact renal function and contribute to various clinical outcomes. The reduction in the number of nephrons results in a decrease in GFR, which in turn affects the health of the kidney and increases the risk of chronic diseases (Figure 3). Mitigating complications and managing geriatric populations necessitate comprehension of these processes [83]. The age-related decrease in GFR measures the extent to which nephron loss occurs during normal aging. According to research, nephrosclerosis, a pathological process associated with aging, is estimated to result in the loss of approximately 6000 to 6500 nephrons in kidneys annually after the age of 30. Healthy adults aged 70–75 years have approximately 48% fewer functioning nephrons than those aged 18–29 years [2]. The gradual loss of nephrons is a critical factor that contributes to a decline in overall renal function, thereby increasing the risk of CKD and other age-related health issues [84]. However, cardiovascular associations appear to vary with age; a recent autopsy study found no association of low birth weight or low nephron number with cardiovascular disease in adults aged ≤55 years, while suggesting that glomerulosclerosis-related nephron loss may contribute to these outcomes later in life [85].

Various physiological and morphological alterations occur in aging kidneys. These include structural changes, such as decreased kidney cortex volume and glomerulosclerosis, associated with the loss of nephrons. Furthermore, the GFR undergoes a gradual decline due to the natural aging process [86]. The burden on the remaining nephrons is increased because of these changes, which impact both the functional capacity of the kidneys and the efficiency of waste removal from the body. The risk of several clinical complications is substantially increased by the decline in nephron number and the associated GFR reduction. Renal insufficiency frequently exacerbates a variety of conditions, such as chronic conditions and metabolic diseases, which are frequently encountered by aging populations [2].

Reduced nephron endowment leads to compensatory glomerular enlargement, hyperfiltration, and increased glomerular pressure. Over time, these adaptations can promote glomerulosclerosis and declining kidney function, increasing the risk of hypertension, cardiovascular disease, chronic kidney disease (CKD), and end-stage kidney disease (ESKD).

Nephrosclerosis is a renal condition characterized by fibrotic changes in the renal vasculature and is recognized as one of the key factors in the development of aging-related renal damage. In this case, structural changes in the blood vessels of the kidneys lead to a loss and reduction in kidney function [87]. This decrease in kidney function can usually be measured by a decrease in GFR, which is a measure of kidney function. The GFR is directly affected by the number of nephrons [86]. Even in old age, although kidney function gradually declines, hypertrophy or enlargement of the remaining glomeruli usually does not show itself in the advanced stages of aging. This means that while some glomeruli may become more stressed due to the additional load from other damaged nephrons, a hypertrophic response is generally not observed in the remaining glomeruli, which is linked to a complex clinical process that depends on it. It affects the quality of life of the elderly [88].

The development of effective strategies for managing renal health in the elderly depends on understanding the mechanisms that underlie nephron decline and aging. Timely implementation of interventions that can maintain the function of the remaining nephrons and increase the quality of life of patients can be achieved through early detection of kidney function decline. Future research should focus on identifying biomarkers that can predict nephron loss and developing guidelines for monitoring renal health in elderly populations [89].

## 10. Nephron Endowment on Hypertension

Low nephron endowment can increase susceptibility to hypertension through reduced capacity to regulate sodium and fluid balance and compensatory changes in surviving nephrons [21,76]. Increased SNGFR and glomerular pressure can sustain filtration initially, but may also increase susceptibility to injury [3,18]. Systemic hypertension can further elevate glomerular stress, accelerating nephron loss and CKD (Figure 1) [27,90,91].

Human and animal studies link adverse developmental conditions and reduced nephron number to elevated blood pressure later in life [42,49,58]. Adult mice with ureteric bud-specific *Fgfr2* deletion develop hypertension with glomerular and tubular injury [92]. Differences among populations require interpretation in the context of genetic, developmental, and environmental influences rather than nephron number alone [27,93,94]. Weight management, dietary sodium restriction, and appropriate antihypertensive treatment are important approaches to reducing cardiovascular and kidney risk [95].

Further research is needed to define when low nephron endowment leads to hypertension, and which individuals benefit most from early intervention. Integrating developmental history, kidney function, albuminuria, and blood pressure may improve risk assessment and guide strategies to preserve kidney function [23,24,81,83].

## 11. Conclusions

The therapeutic strategies that are developed to mitigate nephron loss in CKD are undergoing a rapid evolution, incorporating lifestyle modifications, regenerative medicine, and pharmacological interventions. These strategies prioritize a comprehensive and multifaceted approach to effectively address nephron loss, thereby improving patient outcomes. Future perspectives suggest an optimistic landscape in which advanced research, particularly in genetic and regenerative technologies, will likely result in more targeted and effective treatments for CKD [96]. The most recent developments in pharmacological treatments are essential for the treatment of nephron loss in CKD. The efficacy of medications such as finerenone, a non-steroidal mineralocorticoid receptor antagonist, in reducing the progression of CKD and enhancing cardiovascular outcomes has been demonstrated [97,98]. Furthermore, the potential advantages of targeted pharmacological are underscored by therapies such as semaglutide, a glucagon-like peptide-1 receptor agonist, which have substantially reduced cardiovascular risks and kidney complications in patients with type 2 diabetes and CKD [97].

Using techniques such as tissue engineering and stem cell therapy, regenerative medicine has significant potential to combat nephron depletion. By creating kidney tissues from stem cells to replace damaged nephrons, it is possible to stop the progression of damage, reduce dependence on immunosuppressive treatments, and solve the problem of organ shortage. Despite the significant progress made in this field, continuous efforts are being made to increase the efficiency and scalability of these techniques for clinical applications [99].

Lifestyle modifications are essential to mitigate the effects of nephron loss and manage CKD. Dietary modifications, regular physical activity, and weight management can substantially reduce CKD risk factors, including diabetes and hypertension, promoting renal health. Lifestyle interventions not only improve patient well-being but also have the potential to halt the progression of nephron loss [100]. The future of CKD treatment is contingent upon the adoption of precision medicine and the comprehension of its fundamental genetic determinants [96]. Novel genetic profiles are being discovered through innovative research that utilizes genomics and ‘omics analyses. This information may be used to develop targeted therapies that are specifically designed for specific patient subpopulations. Moreover, the testing and development of new therapies for CKD are anticipated to be revolutionized by advancements in preclinical models, including organs-on-chips and 3D bioprinting, which will emphasize a personalized approach to treatment [101,102]. To effectively address nephron loss in CKD, a comprehensive approach that incorporates pharmacological treatments, regenerative techniques, lifestyle interventions, and genetic research is necessary. Future therapies are designed to enhance the quality of life for CKD patients by not only slowing the progression but also potentially reversing or repairing renal damage, utilizing advancements in a variety of scientific disciplines [103].

## Data Availability

No new data were created or analyzed in this study.

## Abbreviations

The following abbreviations are used in this manuscript:

GFR	glomerular filtration rate
CKD	chronic kidney disease
SNGFR	single nephron glomerular filtration rate
CFE-MRI	cationic ferritin-enhanced magnetic resonance imaging
IUGR	intrauterine growth restriction
GDNF/RET	RET receptor tyrosine kinase via a GFRα co-receptor
FGF	fibroblast growth factors
PAX2	paired box gene 2
HH	hereditary hemochromatosis
ESKD	end-stage kidney disease
